# Alginate Alleviates Dextran Sulfate Sodium-Induced Colitis by Promoting Bifidobacterium animalis and Intestinal Hyodeoxycholic Acid Synthesis in Mice

**DOI:** 10.1128/spectrum.02979-22

**Published:** 2022-10-11

**Authors:** Yu Pi, Xiangyu Zhang, Yujun Wu, Zhenyu Wang, Yu Bai, Xiaoyi Liu, Dandan Han, Jinbiao Zhao, Isabel Tobin, Jiangchao Zhao, Guolong Zhang, Junjun Wang

**Affiliations:** a State Key Laboratory of Animal Nutrition, College of Animal Science and Technology, China Agricultural University, Beijing, China; b Key Laboratory of Feed Biotechnology of Ministry of Agriculture and Rural Affairs, Institute of Feed Research, Chinese Academy of Agricultural Sciences, Beijing, China; c Department of Animal Science, Division of Agriculture, University of Arkansas, Fayetteville, Arkansas, USA; d Department of Animal and Food Sciences, Oklahoma State University, Stillwater, Oklahoma, USA; Jilin University

**Keywords:** alginate, bile acid, hyodeoxycholic acid, inflammatory bowel disease, microbiome

## Abstract

Alginate (ALG) is known to alleviate intestinal inflammation in inflammatory bowel disease, but its mechanism of action remains elusive. In the present study, we studied the involvement of the intestinal microbiota and bile acid (BA) metabolism in ALG-mediated anti-inflammatory effects in mice. A combination of 16S rRNA gene amplicon sequencing, shotgun metagenomic sequencing, and targeted BA metabolomic profiling was employed to investigate structural and functional differences in the colonic microbiota and BA metabolism in dextran sulfate sodium (DSS)-treated mice with or without dietary supplementation of ALG. We further explored the role of the intestinal microbiota as well as a selected ALG-enriched bacterium and BA in DSS-induced colitis. Dietary ALG alleviated DSS-mediated intestinal inflammation and enriched a small set of bacteria including Bifidobacterium animalis in the colon (*P < *0.05). Additionally, ALG restored several bacteria carrying secondary BA-synthesizing enzymes such as 7α-hydroxysteroid dehydrogenase and BA hydrolase to healthy levels in DSS-treated mice. Although a majority of BAs were suppressed by DSS, a few secondary BAs such as hyodeoxycholic acid (HDCA) were markedly enriched by ALG. Furthermore, ALG significantly upregulated the expression of a major BA receptor, the farnesoid X receptor, while suppressing NF-κB and c-Jun N-terminal kinase (JNK) activation. Depletion of the intestinal microbiota completely abrogated the protective effect of ALG in DSS-treated mice. Similar to ALG, *B. animalis* and HDCA exerted a strong anti-inflammatory effect in DSS-induced colitis by downregulating inflammatory cytokines (interleukin-1β [IL-1β], IL-6, and tumor necrosis factor alpha [TNF-α]). Taken together, these results indicated that ALG achieves its alleviating effect on intestinal inflammation through regulation of the microbiota by enriching *B. animalis* to promote the biosynthesis of specific secondary BAs such as HDCA. These findings have revealed intricate interactions among the intestinal microbiota, BA metabolism, and intestinal health and further provided a novel strategy to improve intestinal health through targeted manipulation of the intestinal microbiota and BA metabolism.

**IMPORTANCE** ALG has been shown to ameliorate inflammatory bowel disease (IBD), but little is known about the mechanism of its anti-inflammatory action. This study was the first to demonstrate that ALG provided a preventive effect against colitis in an intestinal microbiota-dependent manner. Furthermore, we confirmed that by selectively enriching intestinal *B. animalis* and secondary BA (HDCA), ALG contributed to the attenuation of DSS-induced colitis. These findings contribute to a better understanding of the mechanism of action of ALG on the attenuation of colitis and provide new approaches to IBD therapy by regulating gut microbial BA metabolism.

## INTRODUCTION

Inflammatory bowel disease (IBD) is a group of disorders that cause chronic intestinal inflammation (1). Accumulating evidence has linked IBD to intestinal microbiota dysbiosis (2, 3). Manipulation of the intestinal microbiota and its metabolites has shown potential in IBD therapy (4–6). However, the specific involvement of intestinal microbes and metabolites in the pathogenesis of IBD remains elusive.

Primary bile acids (BA) are produced in the liver and deconjugated and transformed by the intestinal microbiota to secondary BAs such as deoxycholic acid (DCA) and hyodeoxycholic acid (HDCA) in the intestinal tract (7, 8). Certain strains of *Lactobacillus*, *Erysipelotrichaceae*, *Lachnospiraceae*, *Clostridium*, and *Bacteroides* carry bile salt hydrolase (*bsh*), which is responsible for deconjugation of glycine or taurine from primary BAs, while other strains of *Lactobacillus*, *Lachnospiraceae*, *Ruminococcaceae*, *Clostridiaceae*, *Eubacterium*, and *Peptostreptococcus* carry the BA-inducible (*bai*) gene operon responsible for 7α-dehydroxylation and biotransformation to generate secondary BAs (9–11). Deconjugation and biotransformation of BAs were recently shown to be impaired in IBD patients (12). However, a mechanistic understanding of the microbiota-BA-IBD association is lacking. Thus, it is essential to elucidate the changes in the intestinal microbiome and BA metabolism in the context of intestinal inflammation in order to devise effective therapeutic strategies for IBD (13).

Alginate (ALG), a linear polymer of α-l-guluronic acid and β-d-mannuronic acid, is a natural water-soluble polysaccharide extracted from brown seaweed. ALG is known to be protective against gastric ulcers and esophageal reflux (14, 15). It is also beneficial for ameliorating experimental colitis in mice (16–18). However, poor absorption of ALG by the intestinal tract implies microbial involvement in the metabolism of ALG (19). Consistently, dietary supplementation of ALG increased the bsh enzyme activity and total BA levels and altered the BA profile in the feces of rats (20–22). However, the role of ALG in microbial BA metabolism in the context of intestinal inflammation remains largely unknown.

We hypothesized that ALG alleviates intestinal inflammation by targeting specific intestinal bacteria and BAs. To test this hypothesis, a DSS-induced colitis model was employed to investigate structural and functional differences in the intestinal microbiota and BAs in response to dietary supplementation of ALG. We further selected a bacterium and BA that were highly differentially enriched by ALG and explored their role in DSS-induced intestinal inflammation. This study has shed light on ALG-mediated regulation of the intestinal microbiota and BA metabolism and provided new approaches to the prevention and treatment of IBD.

## RESULTS

### Alginate alleviates DSS-induced intestinal inflammation.

**FIG 1:**
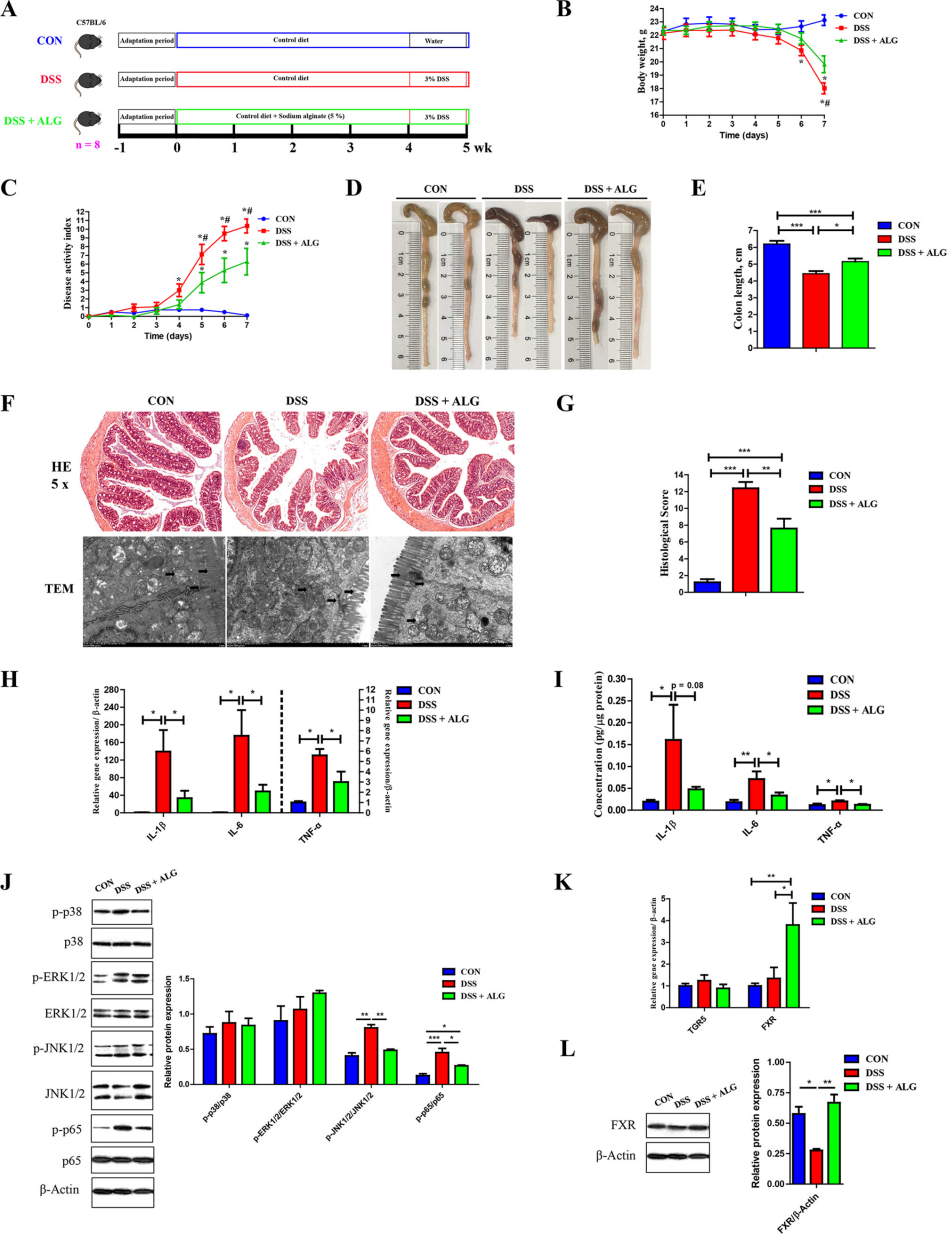
Dietary supplementation of alginate (ALG) alleviates DSS-induced colitis. (A) Experimental scheme of ALG supplementation in a mouse model of DSS-induced acute colitis. (B and C) While DSS (3%) was provided in drinking water for a week to induce colitis, body weight (B) and the disease activity index (DAI) score (C) were recorded daily in the final week of DSS treatment for three groups of mice (*n* = 8). The colon was collected from each mouse at the end of the experiment. (D to G) Representative images of the colon (D), the colon length (E), H&E staining (magnification of ×50), and TEM of the colon (F) and colonic histology score (G) are shown. (H and I) The mRNA (H) and protein (I) expression levels of three inflammatory cytokines in the colon were also measured. (J) Phosphorylation of p38, ERK1/2, JNK1/2, and p65 NF-κB in the colon. (K) The mRNA gene expression levels of two BA receptors in the colon. (L) Expression of FXR at the protein level. Data are presented as means ± SEM. *, *P* < 0.05; **, *P* < 0.01; ***, *P* < 0.001; #, *P* < 0.05; compared with the DSS plus ALG group.

To study the influence of ALG on intestinal inflammation, we supplemented mice with or without 5% ALG in the diet and then subjected them to 3% DSS in their drinking water to induce colitis (Fig. 1A). ALG significantly ameliorated DSS-induced colitis (*P < *0.05), as evidenced by an improvement in weight loss (Fig. 1B), disease activity index (DAI) score (Fig. 1C), and colonic shortening (Fig. 1D and E). Consistently, ALG supplementation showed significant alleviation of mucosal inflammatory cell infiltration and epithelial layer destruction in DSS-treated mice (*P < *0.05) (Fig. 1F and G). Additionally, the mRNA and protein expression levels of proinflammatory cytokines (interleukin-1β [IL-1β], IL-6, and tumor necrosis factor alpha [TNF-α]) in the colon of DSS-treated mice were significantly suppressed by ALG (*P < *0.05) (Fig. 1H and I). Moreover, DSS-induced phosphorylation of c-Jun N-terminal kinase 1/2 (JNK1/2) and NF-κB p65 in the colon was significantly decreased in response to ALG; however, phosphorylation was unchanged with p38 mitogen-activated protein kinase (MAPK) and extracellular signal-regulated kinase 1/2 (ERK1/2) (Fig. 1J).

G-protein-coupled receptor (TGR5) and farnesoid X receptor (FXR) are two major receptors for BAs (23). *TGR5* mRNA was not affected by DSS or ALG, while *FXR* mRNA was significantly induced by ALG in the colon (Fig. 1K). At the protein level, DSS suppressed FXR expression, but it was largely restored by ALG (Fig. 1L). These results indicated that dietary ALG is capable of alleviating intestinal inflammation, possibly by suppressing the NF-κB and JNK signaling pathways.

**FIG 2:**
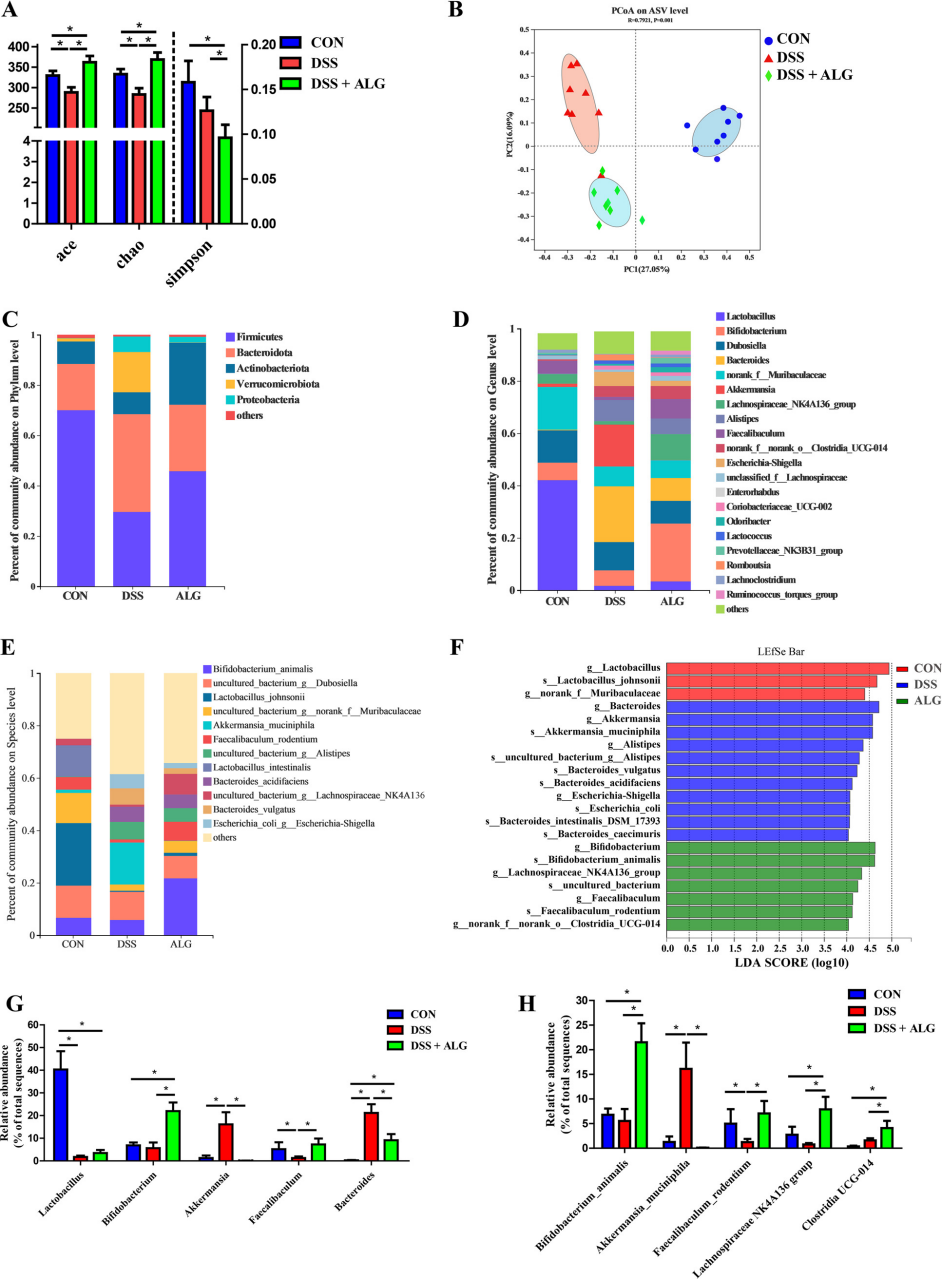
Dietary supplementation with ALG alters the composition of the intestinal microbiota in DSS-treated mice. Mice supplemented with or without 5% ALG for 4 weeks, followed by induction of colitis with 3% DSS in drinking water for another week (*n* = 8). The colonic digesta was collected from each mouse at the end of the experiment and subjected to DNA isolation and 16S rRNA gene sequencing. (A) The α-diversity of the colonic microbiota among three groups of mice. (B) PCoA plot depicting β-diversity of the colonic microbiota based on the Bray-Curtis distance. (A to C) The colonic microbiota composition is shown at the phylum (C), genus (D), and ASVs levels (E). (F) LEfSe analysis of differential enrichment of the colonic bacteria at the ASV level (linear discriminant analysis [LDA] > 4). (G and H) Bar graphs of the top five differentially enriched bacteria are shown at the genus (G) and ASV levels (H). Data are presented as means ± SEM. *, *P* < 0.05.

Alginate selectively promotes Bifidobacterium animalis and alters the microbial metabolic function in DSS-induced colitis. To examine the changes in the intestinal microbiota of DSS-treated mice in response to ALG, we analyzed the colonic microbiota composition using 16S rRNA gene sequencing. While ALG restored DSS-induced loss in the richness of the microbiota as indicated by ACE and Chao1, overall α-diversity was significantly increased in DSS-treated mice in response to ALG as indicated by the Simpson index (*P < *0.05) (Fig. 2A). Principal-coordinate analysis (PCoA) based on the Bray-Curtis distance showed clear segregation among the three groups of mice (Fig. 2B). The colonic microbiota composition was different at the phylum (Fig. 2C), genus (Fig. 2D), and amplicon sequence variant (ASVs) levels (Fig. 2E). The linear discriminant analysis effect size (LEfSe) algorithm further identified several ASVs that were differentially abundant among three groups of mice (Fig. 2F). The relative abundance of *Lactobacillus* in the colon was significantly suppressed by DSS but failed to be recovered by ALG (Fig. 2G). *Bacteroides*, on the other hand, was markedly expanded in response to DSS but suppressed by ALG (Fig. 2G). It is important to note that Bifidobacterium animalis, a highly abundant bacterium in the colon, was drastically increased from 6.79% in healthy or DSS-treated mice to 21.5% in the DSS plus ALG mice (Fig. 2H). ALG also markedly induced *Lachnospiraceae* NK4A136 group and *Clostridia* UCG-014 in both control and DSS-treated mice and restored Faecalibacterium rodentium in DSS-treated mice to the healthy level (Fig. 2H). Interestingly, Akkermansia muciniphila was significantly increased by DSS but almost completely abolished in response to ALG supplementation (Fig. 2H).

**FIG 3:**
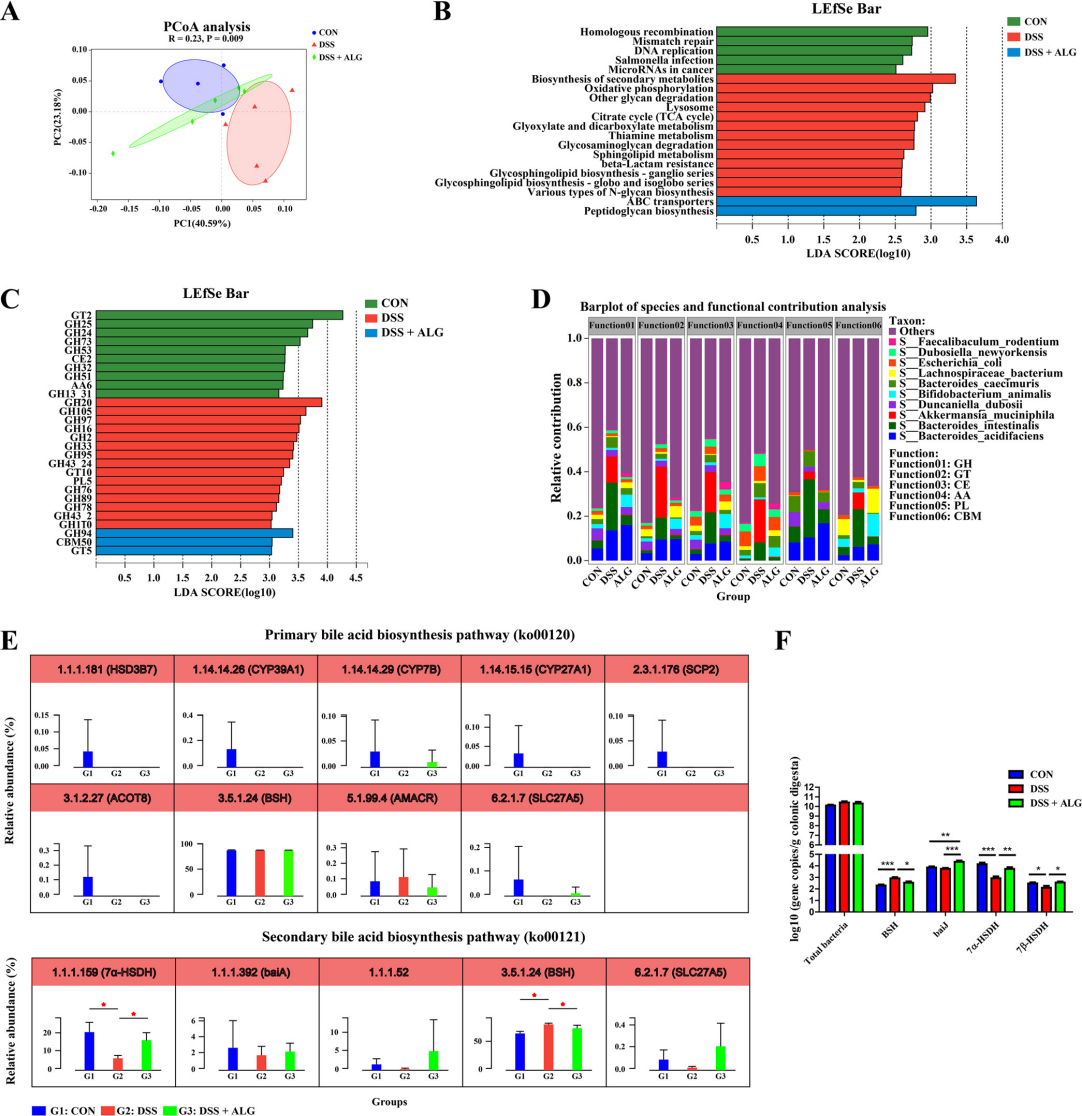
Dietary supplementation with ALG modulates the function of the intestinal microbiota in DSS-treated mice. Mice supplemented with or without 5% ALG for 4 weeks, followed by induction of colitis with 3% DSS in drinking water for another week (*n* = 8). The colonic digesta was collected from each mouse at the end of the experiment and subjected to DNA isolation and shotgun metagenomic sequencing. (A) PCoA plot based on the KEGG pathways of identified bacterial genes. (B) LEfSe analysis of differential enrichment of microbial KEGG pathways (LDA > 2.5). (C) LEfSe analysis of differential enrichment of microbial carbohydrate-active enzymes (CAZymes) (LDA > 3). (D) Functional contribution of top 10 differentially enriched CAZymes. (E) Relative abundances (%) of the major enzymes involved in the biosynthesis of primary and secondary bile acids (BAs). (F) Quantitative PCR analysis of the copy number of total bacteria and specific bacterial groups carrying BA hydrolase (*bsh*), BA 7-dehydroxylase (*baiJ*), 7α-hydroxysteroid dehydrogenase (*7α-HSDH*), and 7β-hydroxysteroid dehydrogenase (*7β-HSDH*). Data are presented as means ± SEM. *, *P* < 0.05; **, *P* < 0.01; ***, *P* < 0.001.

To further understand the functional changes of the colonic microbiota of mice in response to DSS and ALG, shotgun metagenome sequencing was used. The PCoA plot again revealed distinct functional profiles among the three groups based on the KEGG enzymes (analysis of similarity [ANOSIM], *P < *0.05; Fig. 3A). LEfSe analysis showed differential enrichment of multiple metabolic pathways. For example, ATP-binding cassette (ABC) transporters that utilize ATP hydrolysis to provide energy for the translocation of substrates across cell membranes (24) were enriched in the DSS plus ALG group (Fig. 3B). We further identified the enrichment of several carbohydrate-active enzymes (CAZymes) such as glycoside hydrolase 94 (GH94), carbohydrate-binding module 50 (CBM50), and glycosyltransferase 5 (GT5) in the DSS plus ALG group (Fig. 3C). In fact, *B. animalis* was the main contributor (top 3) of these CAZymes (GH, GT, and CBM) (Fig. 3D).

### Alginate alters microbial BA metabolism and increases intestinal HDCA in DSS-induced colitis.

The intestinal microbiota plays a key role in the biotransformation of primary BAs to secondary BAs by deconjugation and dihydroxylation (23). The major enzymes involved in the biosynthesis of primary and secondary BAs are bsh, baiJ, 7α-hydroxysteroid dehydrogenase (7α-HSDH), and 7β-hydroxysteroid dehydrogenase (7β-HSDH) (23). To study the impact of dietary supplementation of ALG on intestinal BA biosynthesis, we examined the changes in the major enzymes involved in the biosynthesis of primary and secondary BAs based on the shotgun metagenomic data. The host enzymes responsible for the biosynthesis of primary BAs were largely unaltered by DSS or ALG (Fig. 3E). Among those major enzymes involved in secondary BA synthesis, 7α-HSDH was significantly suppressed by DSS, while bsh was enriched; however, ALG restored relative abundances of both enzymes to healthy levels (Fig. 3E). Consistently, the quantitative PCR (qPCR) analysis revealed that the bacteria carrying *7α-HSDH*, *7β-HSDH*, and *baiJ* were significantly increased, whereas those carrying *bsh* were decreased in DSS-treated mice in response to dietary ALG (*P < *0.05) (Fig. 3F).

**FIG 4:**
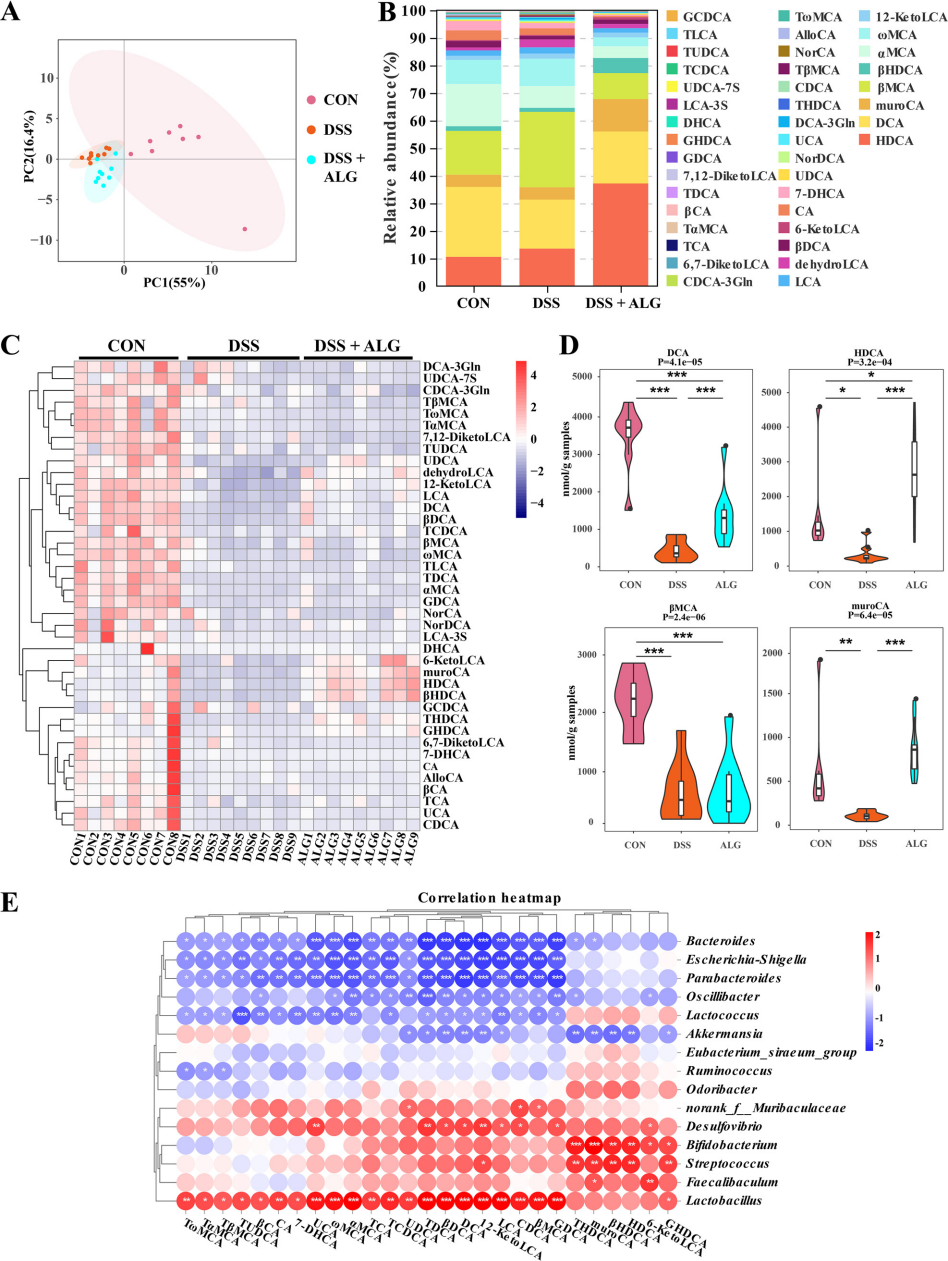
Dietary supplementation with ALG alters the intestinal BA profile in DSS-treated mice. Mice supplemented with or without 5% ALG for 4 weeks, followed by induction of colitis with 3% DSS in drinking water for another week (*n* = 8). The colonic digesta was collected from each mouse at the end of the experiment and subjected to targeted BA profiling. (A) PCA plot of the BA profile among three groups of mice. (B) Relative abundances (%) of different BAs among three groups. (C) Z-score plot of all BAs identified among three groups. (D) Alterations of representative BAs among three groups. (E) Spearman correlation between the relative abundance of the intestinal microbiota and BA concentrations. Data are presented as means ± SEM. *, *P* < 0.05; **, *P* < 0.01; ***, *P* < 0.001.

Targeted BA metabolomics was further employed to examine the effect of ALG on intestinal BAs. Clear separation in the BA profile among the three groups of mice was observed (Fig. 4A). Of 40 identified BAs, HDCA, DCA, β-muricholic acid (βMCA), murocholic acid (muroCA), and α-muricholic acid (αMCA) were the five most abundant BAs in the mouse colon (Fig. 4B). Although a majority of BAs were suppressed by DSS (Fig. 4C), a few BAs, such as DCA and muroCA, were largely restored in response to ALG (Fig. 4D). Notably, HDCA was significantly reduced by DSS but enriched drastically by ALG to become the most abundant BA in the mouse colon (Fig. 4D). Spearman’s correlation analysis further revealed strong positive and negative correlations between many of the significantly altered colonic bacteria and BAs (Fig. 4E). For example, the HDCA concentration was positively correlated with *B. animalis*. Collectively, these results suggested that the anti-inflammatory effect of ALG in DSS-induced colitis may be due to the alteration of intestinal microbial BA metabolism.

### Alginate-mediated alleviation of intestinal inflammation is dependent upon the microbiota.

**FIG 5:**
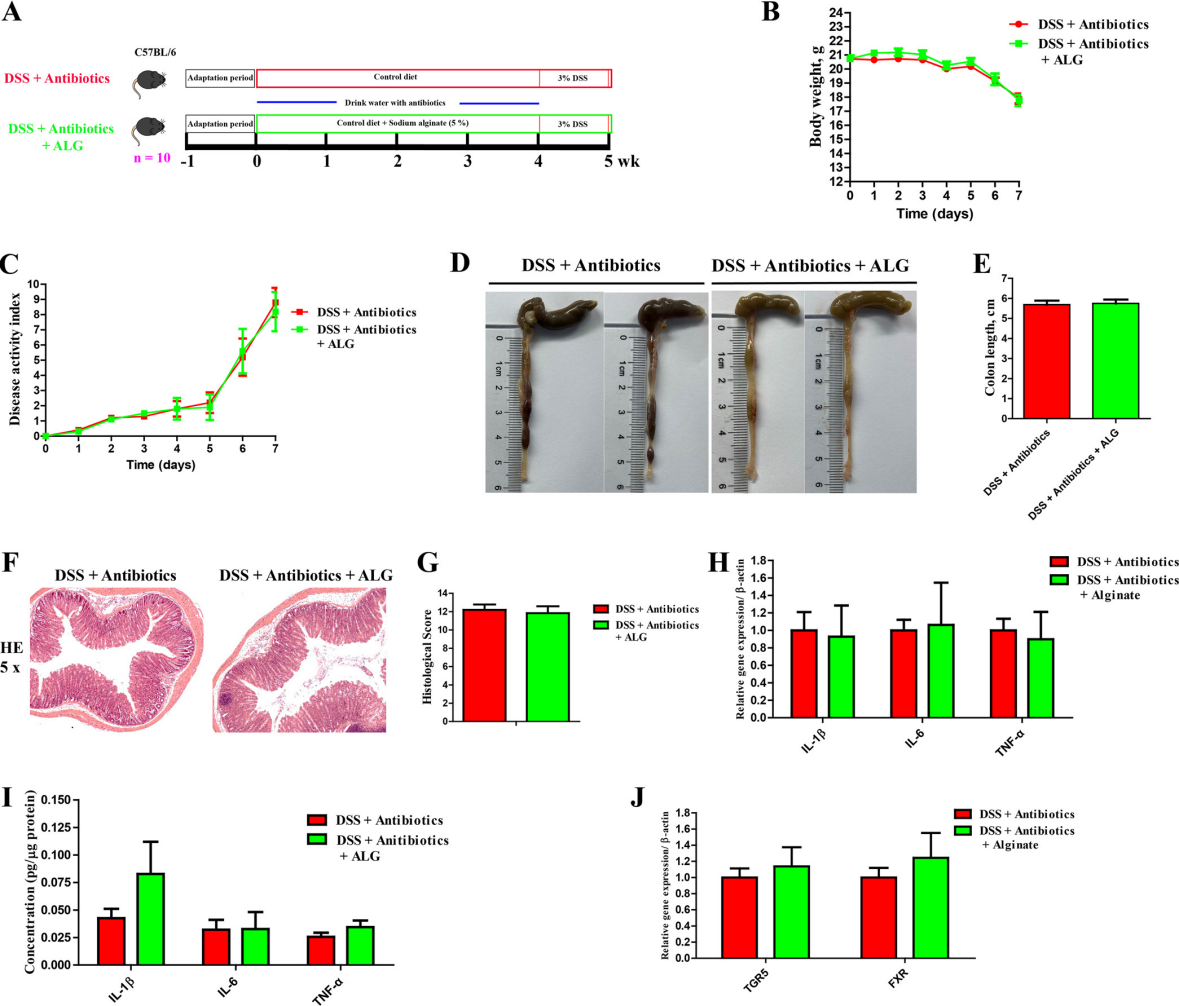
Microbiota depletion abrogates the anti-inflammatory effect of ALG in DSS-treated mice. (A) Experimental scheme to show depletion of the intestinal microbiota by a cocktail of antibiotics and timing of ALG and DSS treatment (*n* = 10). (B and C) Body weight (B) and disease activity index (DAI) score (C) were recorded daily in the final week of DSS treatment for both groups of mice. The colon was collected from each mouse at the end of the experiment. (D to F) Representative images of the colon (D), the colon length (E), H&E staining (magnification, ×50 ) of the colon (F), and colonic histology score (G) are shown. (H and I) The mRNA (H) and protein (I) expression levels of three inflammatory cytokines in the colon were also measured. (J) The mRNA gene expression levels of two BA receptors in the colon.

Because of drastic changes in the intestinal microbiota in DSS-treated mice in response to ALG, we hypothesized that the anti-inflammatory effect of ALG is mediated through the intestinal microbiota. To test this hypothesis, we supplemented ALG to mice with or without depletion of the intestinal microbiota with a cocktail of antibiotics followed by DSS treatment (Fig. 5A). ALG lost its protective effect on weight loss (Fig. 5B), DAI score (Fig. 5C), colonic length (Fig. 5D and E), and colonic integrity (Fig. 5F and G) in the absence of the intestinal microbiota. Consistently, similar mRNA and protein levels of proinflammatory cytokines (IL-1β, IL-6, and TNF-α) in the colon were observed in microbiota-depleted DSS-treated mice with or without ALG supplementation (Fig. 5H and I). The mRNA expression levels of BA receptors (TGR5 and FXR) were also similar between the two groups of mice (Fig. 5J). Collectively, these results suggested that the intestinal microbiota is responsible for mediating the anti-inflammatory effect of ALG in DSS-induced colitis.

### *B. animalis* ameliorates DSS-induced intestinal inflammation in parallel with altered BA metabolism.

**FIG 6:**
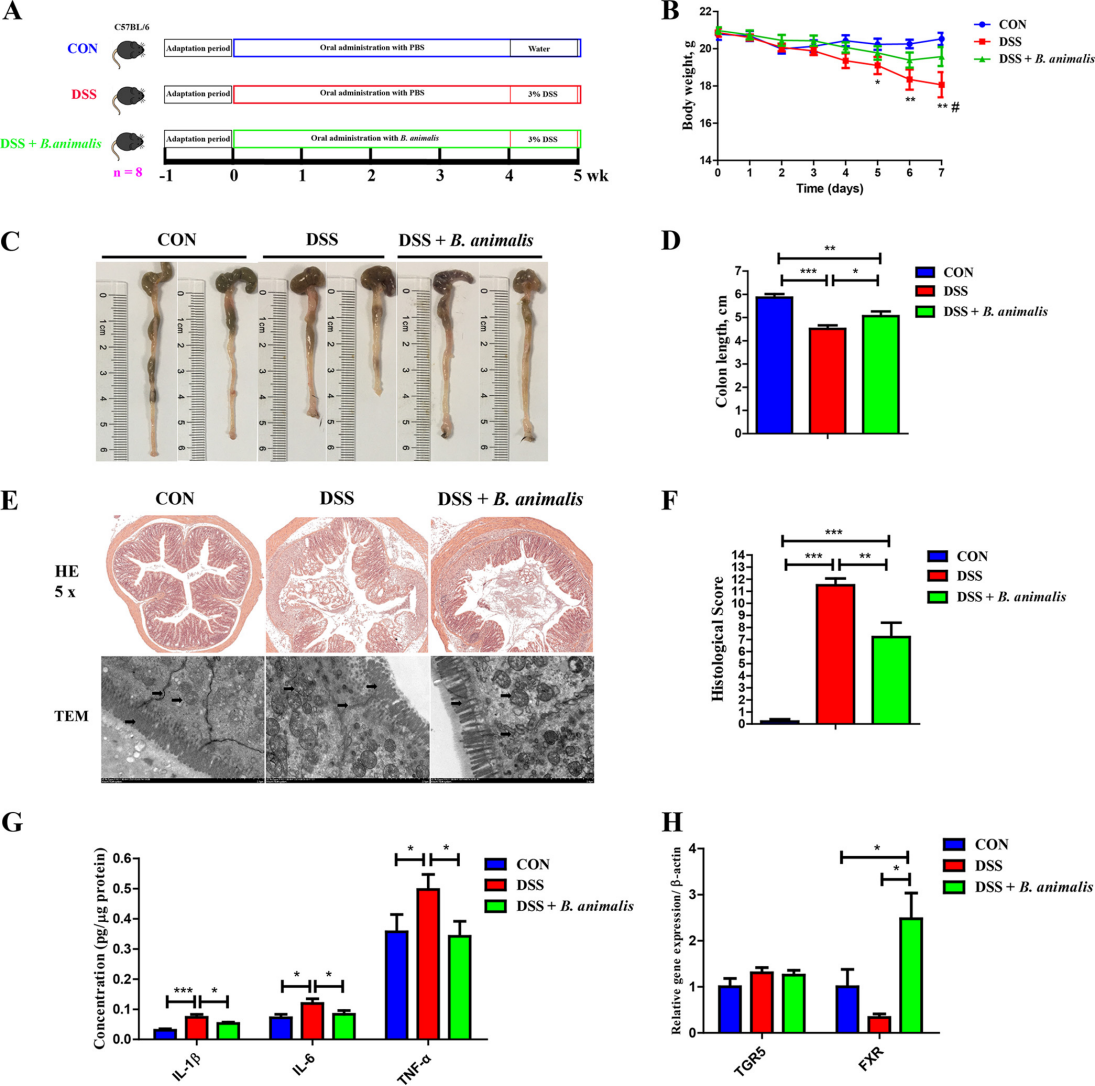
Oral gavage of *B. animalis* alleviates DSS-induced acute colitis in mice. (A) Experimental scheme for DSS-induced colitis and *B. animalis* treatment (*n* = 8). (B) Body weight was recorded daily in the final week of DSS treatment for three groups of mice. The colon was collected from each mouse at the end of the experiment. (C to F) Representative images of the colon (C), the colon length (D), and H&E staining (magnification, ×50) and TEM image of the colon (E) and colonic histology score (F) are shown. (G and H) The mRNA gene expression levels of three inflammatory cytokines (G) and two BA receptors (H) in the colon were also measured by RT-qPCR. Data are presented as means ± SEM. *, *P* < 0.05; **, *P* < 0.01; ***, *P* < 0.001; #, *P* < 0.05; compared with the DSS plus *B. animalis* group.

**FIG 7:**
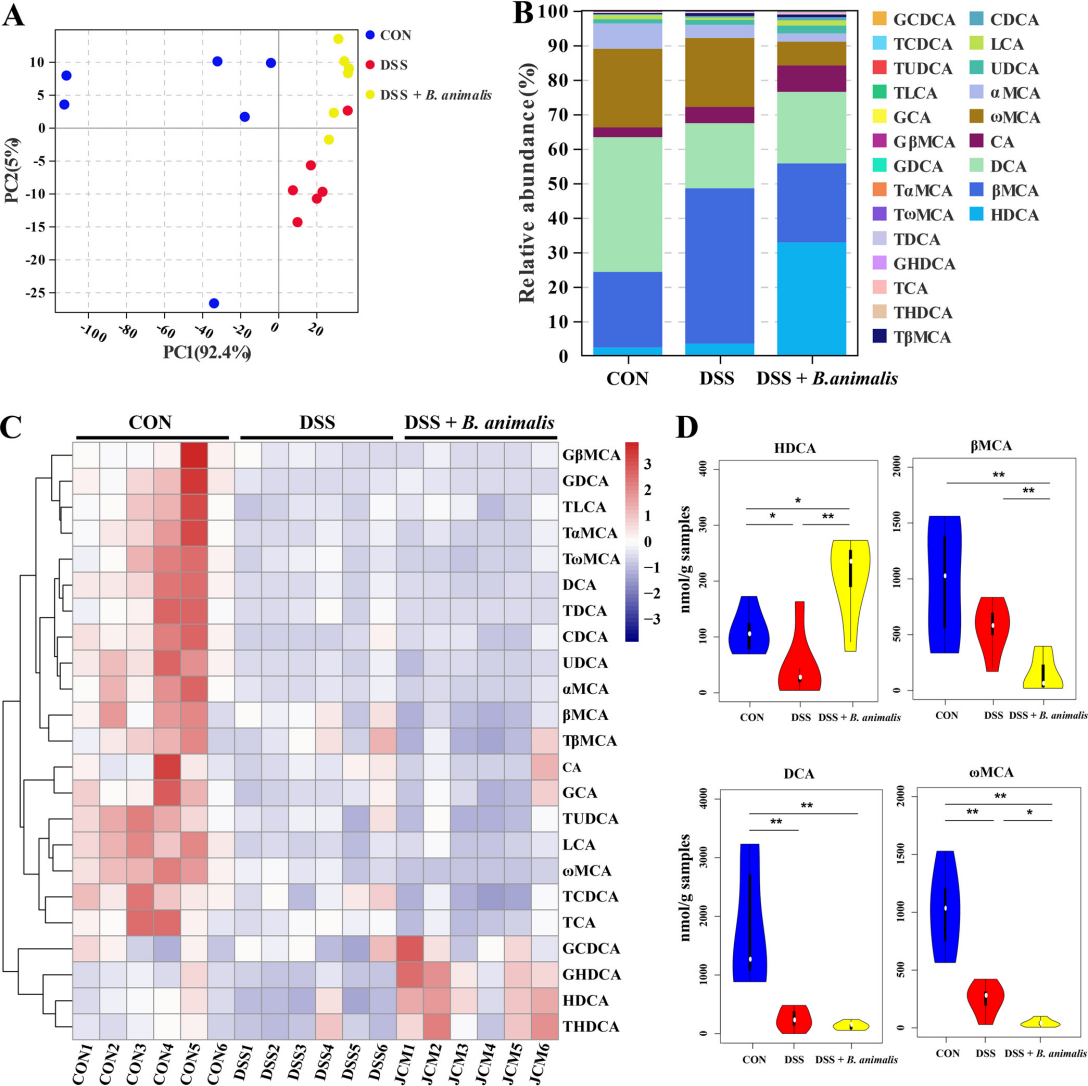
Oral gavage of *B. animalis* alters the intestinal BA profile in DSS-treated mice. Mice supplemented with or without *B. animalis* for 4 weeks, followed by induction of colitis with 3% DSS in drinking water for another week (*n* = 8). The colonic digesta was collected from each mouse at the end of the experiment and subjected to targeted BA profiling. (A) PCA plot of the BA profile among three groups of mice. (B) Relative abundances (%) of different BAs among three groups. (C) Z-score plot of all BAs identified among three groups. (D) Alterations of representative BAs among three groups. Data are presented as the mean ± SEM. *, *P* < 0.05; **, *P* < 0.01.

Since *B. animalis* was the most abundant bacterium that was differentially enriched by ALG in DSS-treated mice, *B. animalis* subsp. *animalis* JCM 1190, which is isolated from mouse feces, was chosen to directly study its anti-inflammatory effect in DSS-treated mice (Fig. 6A). Oral gavage with *B. animalis* significantly ameliorated DSS-induced colitis as evidenced by markedly reducing weight loss (Fig. 6B), colonic shortening (Fig. 6C and D), and epithelial destruction (Fig. 6E and F). Consistently, proinflammatory cytokines (IL-1β, IL-6, and TNF-α) were largely restored to healthy levels in the colon of DSS-treated mice in response to ALG (Fig. 6G). Furthermore, the mRNA expression of FXR in the colon was significantly induced by *B. animalis* (*P < *0.05) (Fig. 6H). *B. animalis* also obviously altered the BA profile in DSS-treated mice (Fig. 7A to 7C). Among the most abundant BAs, HDCA was significantly enhanced by *B. animalis*, while βMCA and ωMCA were significantly reduced (Fig. 7D). Collectively, these results demonstrated that similar to ALG, *B. animalis* is capable of alleviating DSS-induced intestinal inflammation.

### HDCA exerts an anti-inflammatory effect in the DSS-induced colitis mouse model.

**FIG 8:**
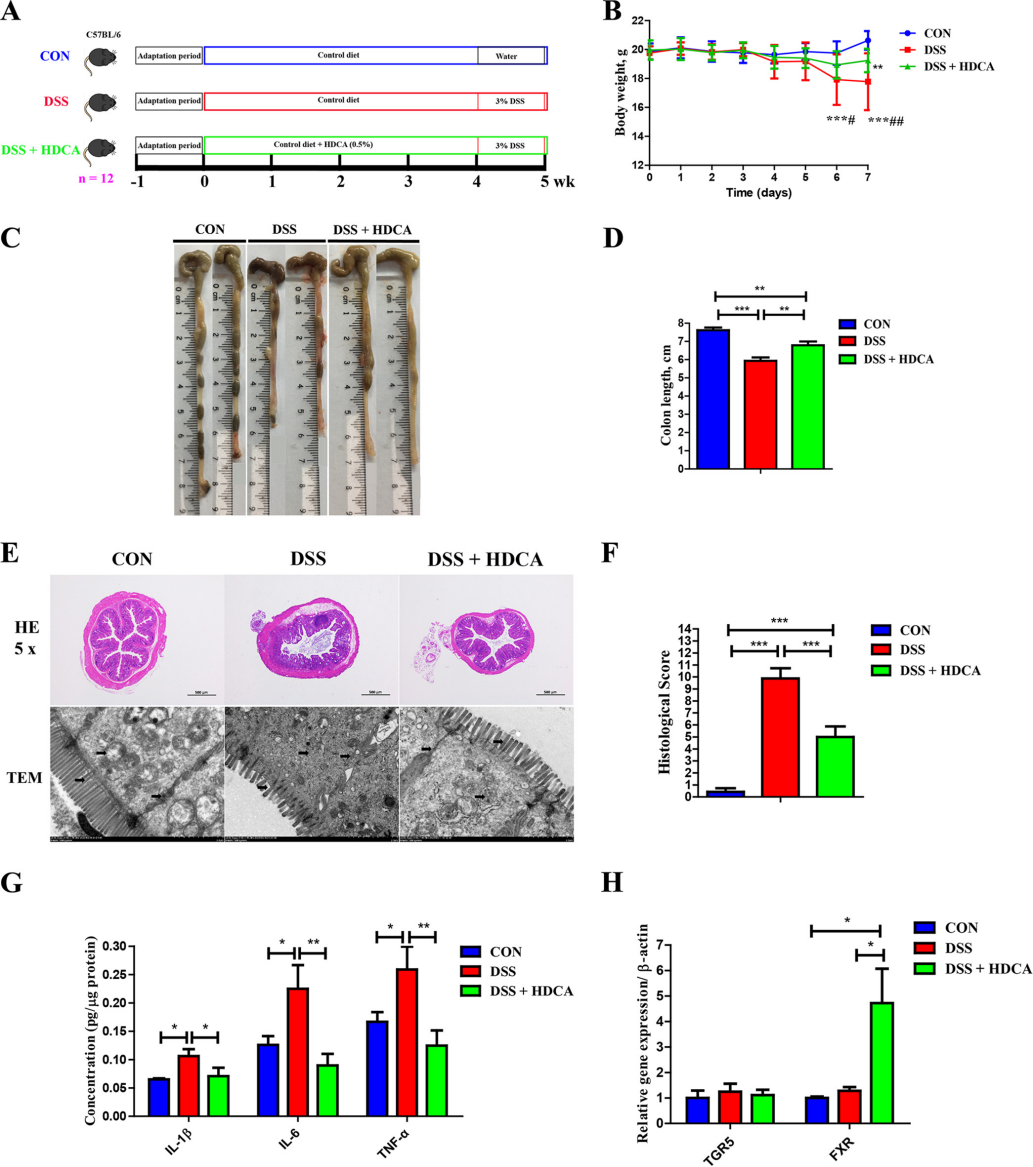
Dietary supplementation with HDCA ameliorates DSS-induced acute colitis. (A) Experimental scheme for DSS-induced colitis and HDCA treatment (*n* = 12). (B) Body weight was recorded daily in the final week of DSS treatment for three groups of mice. The colon was collected from each mouse at the end of the experiment. (C to F) Representative images of the colon (C), the colon length (D), and H&E staining (magnification, ×50), TEM image of the colon (E), and colonic histology score (F) are shown. (G and H) The mRNA gene expression levels of three inflammatory cytokines (G) and two BA receptors (H) in the colon were also measured by RT-qPCR. Data are presented as the mean ± SEM. *, *P* < 0.05; **, *P* < 0.01; ***, *P* < 0.001; #, *P* < 0.05; #*#*, *P* < 0.01; compared with the DSS plus HDCA group.

Because HDCA was drastically increased by ALG and *B. animalis* in DSS-treated mice, we further investigated its role in DSS-induced colitis (Fig. 8A). Dietary supplementation with HDCA significantly improved DSS-induced weight loss (Fig. 8B), colonic shortening (Fig. 8C and D), and mucosal epithelial destruction (Fig. 8E and F). Proinflammatory cytokines (IL-1β, IL-6, and TNF-α) in the colon of DSS-treated mice were restored in response to HDCA (Fig. 8G). Similar to ALG and *B. animalis*, HDCA also significantly increased the mRNA expression of FXR (*P < *0.05) (Fig. 8H). These results suggest that HDCA might be involved in the ALG-mediated anti-inflammatory effect in DSS-induced colitis.

## DISCUSSION

**FIG 9:**
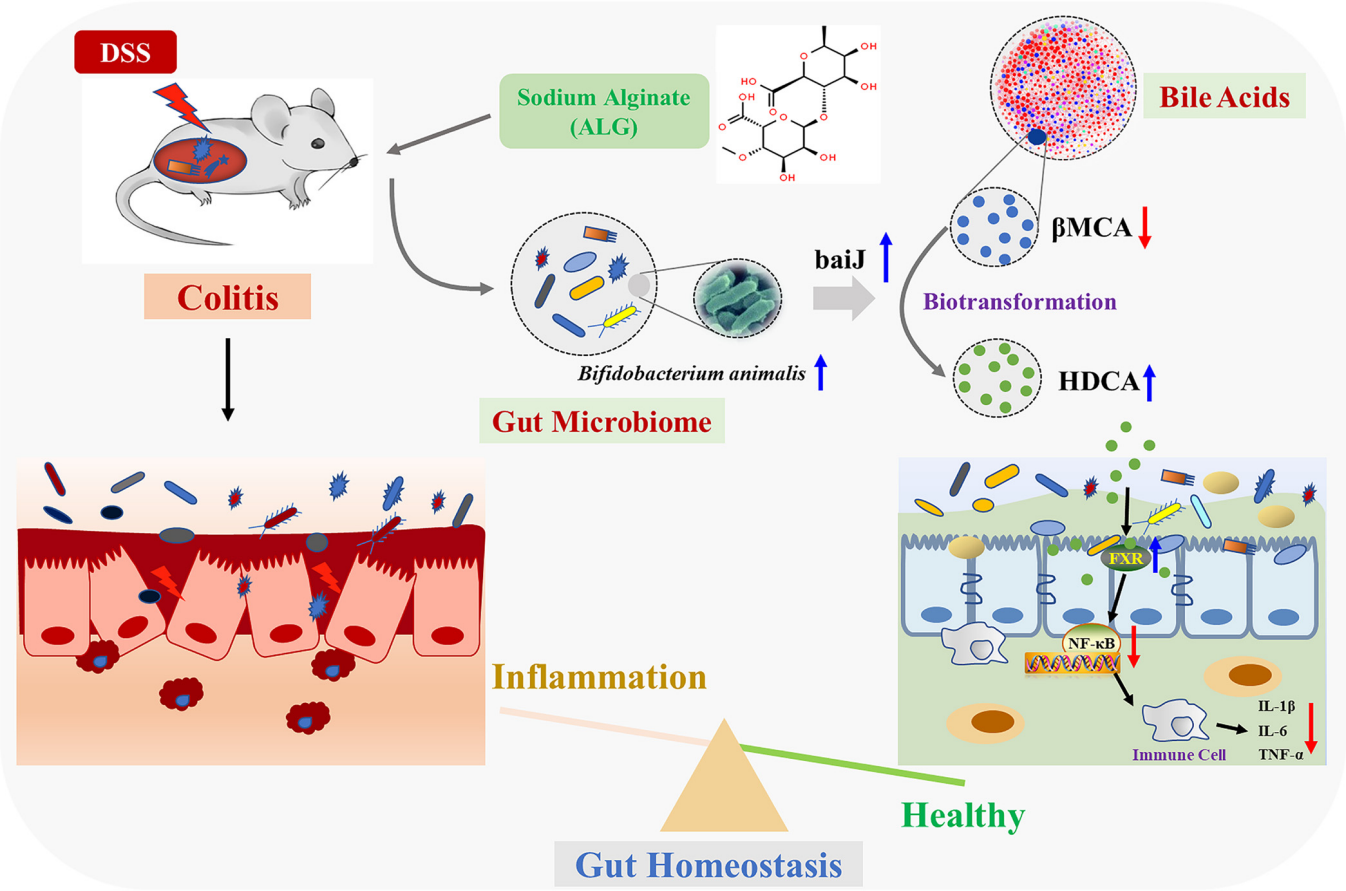
Schematic model showing the potential mechanism of feeding sodium alginate (ALG) that alleviated DSS-induced colitis. Dietary ALG induced an alteration in the gut microbiota to enrich probiotic bacteria such as Bifidobacterium animalis, which subsequently led to an increased production of hyodeoxycholic acid (HDCA), which is biotransformed from β-muricholic acid (βMCA) by BA 7-dehydroxylase (*baiJ*), triggering a cascade of anti-inflammatory responses by activating bile acid receptor, farnesoid X receptor (FXR), and the NF-κB signaling pathway. Ultimately, intestinal epithelial homeostasis is attenuated and colitis was attenuated.

IBD is a chronic inflammatory disorder of the intestinal tract. Although the etiology of IBD remains elusive, the prevailing theory is that IBD results from an exaggerated immune response triggered by dysbiosis and altered microbial BA metabolism in a genetically prone host (12, 25, 26). However, the role of the intestinal microbiota in IBD pathogenesis is unclear. ALG is a soluble dietary fiber known to alter intestinal microbial composition and metabolism (27–29). ALG has been shown to ameliorate experimental IBD in mice (16–18), but little is known about the mechanism of its anti-inflammatory action. In the current study, using a DSS-induced colitis mouse model for IBD, we found that ALG ameliorates IBD-associated intestinal disorder by selectively enriching *B. animalis* and HDCA (Fig. 9).

Of note, microbiota depletion by antibiotics blocks the anti-inflammatory effect of ALG, implying a critical involvement of the intestinal microbiota. In addition, oral administration of *B. animalis* exerts a similar anti-inflammatory effect in DSS-treated mice. Furthermore, HDCA synthesis is also enhanced by *B. animalis* in DSS mice. These results, to the best of our knowledge, have demonstrated for the first time that ALG alleviates intestinal inflammation by modulating intestinal microbiota and microbial BA metabolism. The outcome provides the rationale for using ALG or its enriched bacteria (e.g., *B. animalis*) or bacterial metabolites (e.g., HDCA) for IBD therapy.

In this study, dietary supplementation of low-molecular-weight ALG led to an impressive, greater than a 3-fold expansion of *B. animalis*, which is recognized as a probiotic, making it the most abundant bacterium in the colon (21.5% ± 3.8%) in DSS-treated mice. Consistently, alginate or alginate oligosaccharides have been reported to stimulate the growth of *Bifidobacterium* both *in vitro* and *in vivo* (30, 31). In fact, β-D-mannuronic acid, a component of ALG, is also capable of enriching the intestinal *Bifidobacterium* in mice (32), suggesting that ALG is a preferred carbohydrate source for *B. animalis*, which is consistent with the fact that the genes involved in carbohydrate utilization account for nearly 13% of the genes present in the genome of *Bifidobacterium* (33). In addition, ALG enriches other polysaccharide-utilizing bacteria, including *Lachnospiraceae* NK4A136 group, Faecalibaculum rodentium, and *Clostridia* UCG-014. In agreement, metagenomic analysis has confirmed the enrichment of ABC transporters and CAZymes such as GH94, CBM50, and GT5, indicative of ALG-induced alterations of the microbial function. Moreover, it is also intriguing that in the current study, Akkermansia muciniphila a recognized probiotic, showed a significant increase in the DSS group, which is consistent with previous studies (34, 35). *A. muciniphila* exhibits a modulatory effect on the mucosal immune response in experiments involving both mice and humans and has the potential to be used as a next-generation beneficial microbe (36, 37). *A. muciniphila*, a symbiotic bacterium of the mucus layer, can utilize mucin as its sole carbon, nitrogen, and energy source (37). Thus, we speculate that the increased *A. muciniphila* in the colon of mice with colitis mice may cause, by the large number of intestinal mucosae, shedding induced by inflammation. Whether *A. muciniphila* could exert an adverse effect during DSS-induced colitis still needs further investigation.

It is noteworthy that we observed significant changes in BA metabolism in response to dietary supplementation of ALG. Although a majority of BAs are suppressed by DSS, HDCA is markedly enhanced by ALG. HDCA is believed to be converted from βMCA, a primary BA, by 7α-dehydroxylase (baiJ), which is present in a variety of intestinal bacteria, such as *Lactobacillus*, *Lachnospiraceae*, *Ruminococcaceae*, *Clostridiaceae*, *Eubacterium*, and *Peptostreptococcus* (9–11, 38, 39). We found a reciprocal change of HDCA and βMCA in response to ALG, with the former being significantly increased, while the latter remained suppressed in the colonic digesta of DSS-treated mice. This is perhaps not surprising given the significant enrichment of *Lachnospiraceae* NK4A136 group and *Clostridia* UCG-014 as well as those bacteria encoding the *baiJ* gene in response to ALG. A large amount of HDCA is likely converted from βMCA as a result of an increase in *baiJ* in ALG-supplemented mice. Consistent with our study, a strong correlation was observed earlier between HDCA and a member of *Clostridium* genus (40).

We further demonstrated in this study that ALG supplementation enhances intestinal HDCA synthesis, which is positively correlated with the relative abundance of *B. animalis*. Direct administration of *B. animalis* also similarly promotes the intestinal HDCA concentration, suggesting that *B. animalis* may be a major player in the biosynthesis of HDCA. Collectively, these findings indicated that ALG alleviates intestinal inflammation by modulating the structure and function of the intestinal microbiota. *B. animalis* and HDCA are perhaps major mediators of the anti-inflammatory activity of ALG. In fact, *B. animalis*, Faecalibaculum rodentium, and *Clostridia*_UCG-014 as well as several secondary BAs are known to be anti-inflammatory (41–43). In addition to BAs, short-chain fatty acids are possibly regulated by ALG and may contribute to the beneficial effect of ALG in intestinal inflammation, which warrants further investigation.

HDCA was shown to prevent gallstone formation (44), inhibit atherosclerosis formation (45), and improve glucose homeostasis (46). We found that HDCA significantly downregulates the expression of proinflammatory cytokines (IL-1β, IL-6, and TNF-α) in the colon of DSS-treated mice. Consistent with our findings, HDCA shows an anti-inflammatory effect in neurovascular and microglial cells (47, 48). In addition, dietary supplementation with 2 g/kg of HDCA improves humoral immunity by elevating serum IgA and IgG concentrations in weaned piglets (49). In the current study, HDCA appears to be a major factor in mediating the anti-inflammatory effect of ALG in the intestinal tract.

BAs function through interactions with host BA receptors such as FXR and TGR5 (50). In general, unconjugated BAs are high-affinity ligands of FXR (23). Activation of FXR strongly induces anti-inflammatory genes (51), suppresses NF-κB activation (52), and reduces inflammation in the intestine (53). Conversely, FXR deficiency exacerbates intestinal inflammation in DSS-induced colitis (53). We found in this study that FXR, but not TGR5, is induced by supplementation of ALG, *B. animalis*, and HDCA, suggesting that FXR might be a major receptor that mediates their anti-inflammatory action. Consistently, HDCA suppresses intestinal epithelial cell proliferation via FXR, but not TGR5, in porcine IPEC-J2 cells (54). In addition, dietary supplementation of HDCA significantly enhances intestinal FXR gene expression in weaned piglets (54).

In conclusion, ALG effectively suppresses intestinal inflammation in a DSS-induced colitis model in an intestinal microbiota-dependent manner. ALG preferentially enriches a small set of the bacteria, such as *B. animalis*, which subsequently facilitates the biotransformation of primary BAs to secondary BAs such as HDCA, resulting in NF-κB inhibition and suppression of intestinal inflammation. Our findings contribute to a better understanding of the mechanism of action of ALG and provide new approaches to IBD therapy.

## MATERIALS AND METHODS

### Characterization of sodium alginate.

Sodium alginate (>98%) was obtained from Qingdao Bright Moon Seaweed Group Co., Ltd. (Qingdao, China). The average molecular weight of ALG was determined using gel permeation chromatography (GPC). Briefly, samples were dissolved in NaNO_3_ at 2 mg/mL, and 200 μL was injected in an Agilent 1100 high-pressure liquid chromatography (HPLC) system equipped with organic and aqueous GPC with a flow rate of 1 mL/min and a column thermostat temperature of 35°C. The eluent was monitored by a refractive index detector with an optical unit temperature of 35°C and peak width of 0.1 min. The ratio of α-l-guluronic (G) to β-d-mannuronic acid (M) in ALG was analyzed using infrared spectroscopy with a Mattson 1000 spectrophotometer (Unicam, Cambridge, England). Measurements were recorded of between 400 and 4,000 cm^−1^. ALG used in the present study has a number average molecular weight (M_n_) and weight average molecular weight (*M*_w_) of 8.98 kDa and 21.51 kDa, respectively, and the G/M ratio is 0.69 (see Fig. S1 in the supplemental material).

### Mouse model of DSS-induced colitis.

Female C57BL/6 mice (7 weeks old) were purchased from SPF Biotechnology Co., Ltd. (Beijing, China), and housed in an environmentally controlled room (temperature, 25 ± 2°C; relative humidity, 45 to 60%; lighting cycle, 12 h light [L]:12 h dark [D]) with *ad libitum* access to food and water throughout the studies. All experimental procedures were approved by the Institutional Animal Care and Use Committee of China Agricultural University, Beijing, China.

Acute colitis was induced by the addition of 3% dextran sulfate sodium (DSS, 36 to 50 kDa; MP Biomedicals, Irvine, CA, USA) to drinking water for 7 days. Body weight was measured daily. On day 8, mice were sacrificed, and the entire colon was removed (from the cecum to the anus), and its length was measured. A 2-cm segment of the descending colon was fixed in 4% formalin and processed for hematoxylin and eosin (H&E) staining. Histological scoring was blindly performed by an experienced pathologist as previously described (55). The degree of epithelial loss on the intestinal surface, crypt destruction, and inflammatory cell infiltration were assessed and included in the histopathological examination (Table S1). The severity of colitis was evaluated using a disease activity index (DAI) as described previously (Table S2) (55). The remaining colon samples were stored at −80°C for gene and protein expression analyses. Colonic digesta was also collected and frozen at −80°C for microbial DNA extraction and BA profiling.

### Dietary supplementation of sodium alginate.

After 1 week of acclimation, mice were randomly divided into three groups: a control group (CON), a DSS-treated group (DSS), and a DSS-treated group supplemented with ALG (DSS plus ALG) (*n* = 8). Mice in the CON group were fed the standard diet alone for 5 weeks, while those in the DSS group were fed the standard diet for 5 weeks and colitis was induced with 3% DSS in drinking water in the last week of the experiment. Mice in the DSS plus ALG group were supplemented with 5% ALG for 4 weeks, followed by induction of colitis with 3% DSS in drinking water for another week. The dosage of ALG in the diet was based on previous studies (56, 57). At the end of the experiment, all mice were sacrificed, and postmortem procedures were performed as described above.

### Antibiotic depletion of the intestinal microbiota in mice.

After 1 week of acclimation, mice were provided with a cocktail of antibiotics (1 g/L streptomycin, 0.5 g/L ampicillin, 1 g/L gentamicin, and 0.5 g/L vancomycin) in the drinking water to deplete the intestinal microbiota as previously described (58). All antibiotics were purchased from Meilun Bio (Dalian, China), and drinking water was replenished every other day. Mice were fed the standard diet with or without 5% ALG for 5 weeks, followed by colitis induction in week 5. Antibiotics were provided throughout the duration of the experiment. The postmortem procedures were performed as described above.

### Dietary supplementation of Bifidobacterium animalis.

*B. animalis* subsp. *animalis* JCM 1190 was purchased from BeNa Culture Collection (BNCC 186305, Beijing, China) and cultured in MRS medium. After 1 week of acclimation, mice were orally gavaged with 0.2 mL phosphate-buffered saline (PBS) supplemented with or without 1 × 10^9^ CFU *B. animalis* subsp. *animalis* JCM 1190 every other day for 5 weeks. The dosage of *B. animalis* subsp. *animalis* JCM 1190 was based on a previous study (59). DSS was added to the drinking water in week 5 to induce colitis. At the end of the trial, all animals were sacrificed, and postmortem procedures were performed as described above.

### Dietary supplementation of hyodeoxycholic acid.

HDCA (>98%) was procured from Shanghai Yuanye Bio-Technology Co., Ltd. (Shanghai, China). After 1 week of acclimation, mice were randomly divided into three groups (*n* = 12) fed with a standard diet supplemented with or without 0.5% hyodeoxycholic acid for 5 weeks. Colitis was induced with 3% DSS in drinking water in week 5, except for those mice in the control group. At the end of the trial, all animals were sacrificed, and postmortem procedures were performed as described above.

### Transmission electron microscopy (TEM) analysis.

A 5-mm segment of fresh colonic tissues was flushed with PBS, fixed in 2.5% glutaraldehyde at 4°C for 4 h, and fixed in 1% osmium tetroxide for 2 h at room temperature. Tissues were embedded in pure EMBed 812 (SPI Supplies, West Chester, PA, USA) at 37°C overnight, processed, and stained sequentially in 2% uranium acetate saturated alcohol solution to avoid light staining for 8 min, rinsed in 70% ethanol 3 times, and then rinsed in ultrapure water 3 times; 2.6% lead citrate was used to avoid CO_2_ staining for 8 min and then rinsed with ultrapure water 3 times. After being dried with the filer paper, the cuprum grids were put into the grid board and dried overnight at room temperature. Images were acquired using a transmission HT7700 electron microscope (Hitachi, Tokyo, Japan).

### RNA extraction and Real-Time Quantitative PCR (RT-qPCR).

Total RNA was extracted from a segment of the colon using TRIzol (TaKaRa Bio, Otsu, Shiga, Japan), and 1 μg RNA was reverse-transcribed using a PrimeScript RT reagent kit with gDNA Eraser (TaKaRa Bio Inc, Dalian, China). Quantitative PCR (qPCR) was performed with gene-specific primers (Table S3) on an ABI 7300 real-time PCR system (Applied Biosystems, Foster, CA, USA) with SYBR green master mix (TaKaRa Bio) as previously described (60). Relative changes in mRNA expression were analyzed using the ΔΔ*CT* method as previously described (60).

### Enzyme-linked immunosorbent assay (ELISA).

Frozen colon samples were ground into powder in liquid nitrogen and lysed in RIPA buffer (150 mM NaCl, 1% Triton X-100, 0.5% sodium deoxycholate, 0.1% SDS, 50 mM Tris-HCl, pH 7.4) containing a cocktail of protease inhibitors (Thermo Fisher Scientific, Rockford, IL, USA). After centrifugation at 13,000 × *g* for 10 min at 4°C, the protein concentration of each supernatant was measured using a bicinchoninic acid (BCA) protein assay kit (Beyotime Biotechnology, Beijing, China). The concentrations of IL-1β, TNF-α, and IL-6 in the supernatant were measured the with respective ELISA kits (Thermo Fisher Scientific) according to the manufacturer’s instructions.

### Western blot analysis.

An equal amount of protein (30 μg) from each colonic sample was electrophoresed on SDS polyacrylamide gels with prestained protein markers. Proteins were then transferred to polyvinylidene difluoride (PVDF) membranes (Millipore, Billerica, MA, USA) and blocked in 5% fat-free powdered milk at room temperature in Tris-Tween-buffered saline (TTBS: 20 mM Tris/150 mM NaCl, pH 7.5, and 0.1% Tween 20) for 1 h. The membranes were incubated separately with the following primary antibodies at 4°C overnight with gentle rocking (all from Cell Signaling Technology, Danvers, MA, USA): FXR (catalog [cat.] no. 72105, 1:1,000), p65 (cat. no. 6956, 1:1,000), p-p65 (cat. no. 3033, 1:1,000), p38 (cat. no. 8690, 1:1,000), p-p38 (cat. no. 4511, 1:1,000), ERK1/2 (cat. no. 4695, 1:1,000), p-ERK1/2 (cat. no. 4370, 1:2,000), JNK1/2 (cat. no. 9252, 1:1,000), and p-JNK1/2 (cat. no. 4668, 1:1,000). Membranes were then washed and incubated with a horseradish peroxidase-conjugated secondary antibody (goat anti-rabbit IgG; Thermo Fisher Scientific) at a 1:5,000 dilution for 1 h at room temperature. Signals were developed with SuperSignal West Dura extended duration substrate (Pierce, Rockford, IL, USA) and quantified on an Alpha Imager 2200 (Alpha Innotech Corporation, California, USA). The signal intensities were normalized to β-actin.

### Microbial DNA extraction, 16S rRNA gene sequencing, and data analysis.

Total microbial genomic DNA in the colonic digesta was extracted using a QIAamp fast DNA stool minikit (Qiagen, Germany). The V3 to V4 regions of the 16S rRNA gene were amplified with primers 341F (5′-ACTCCTACGGGAGGCAGCAG-3′) and 806R (5′-GGACTACHVGGGTWTCTAAT-3′) as previously described (61). PCR products were purified, quantified, and pooled at equal amounts for PE300 sequencing on an Illumina MiSeq instrument (Illumina, San Diego, CA, USA) according to the standard protocols of Majorbio Bio-Pharm Technology Co., Ltd. (Shanghai, China). Raw sequences were analyzed using QIIME 2 (version 2020.2) (62) on the Majorbio I-Sanger cloud platform (https://cloud.majorbio.com/). Quality control and denoising were performed using DADA2 under default settings to generate ASVs (63). Only those ASVs with a minimum of two reads and present in more than one sample were retained. A phylogenetic tree was generated using the SEPP algorithm against the Silva 138 database under default settings (64). The α- and β-diversities were calculated using vegan (version 3.3.1). PCoA was performed using Bray-Curtis distance metrics. Differential taxa were identified using the LEfSe (linear discriminant analysis effect size) algorithm and further confirmed using BLAST against the NCBI 16S rRNA database (63). The functional potential of the intestinal microbiota was predicted using PICRUSt2 (65). The raw reads were deposited in the NCBI Sequence Read Archive (SRA) database (accession number SRP303220). In addition, the total bacterial and functional microbial genes, including *baiJ*, *bsh*, *7α-HSDH*, and *7β-HSDH*, involved in the conversion of primary to secondary BAs were quantified using qPCR and bacterial group-specific primers (Table S3) on an ABI 7300 real-time PCR system (Applied Biosystems, Foster City, CA, USA) with SYBR green master mix (TaKaRa Bio) (60).

### DNA extraction, library preparation, and metagenomic sequencing.

Colonic digesta samples were also subjected to shotgun metagenomic sequencing. Briefly, microbial genomic DNA was extracted using the E.Z.N.A soil DNA kit (Omega Bio-Tek, Norcross, GA, USA). DNA concentration was measured with a TBS-380 fluorometer (Promega, Madison, WA, USA). The quality of extracted DNA was checked on 1% agarose gel. DNA was then fragmented to approximately 400 bp using a Covaris M220 ultrasonicator (Gene Company, China). Adapter ligation, cleanup, and enrichment were performed using a NEXT-FLEX rapid DNA sequencing (DNA-seq) kit (Bioo Scientific, Austin, TX, USA). The library was analyzed for size distribution, quantified using qPCR, and sequenced PE150 on an Illumina NovaSeq 6000 device at a depth of greater than 10 Gb raw reads/sample.

### Metagenome assembly and functional annotation.

Shotgun sequencing reads were analyzed on the online platform of Majorbio Cloud platform (www.majorbio.com). Quality control was performed using fastp (version 0.20.0) to trim adapters and filter low-quality reads with the parameter –cut_by_quality3 -W 4 -M 20 -n 5 -c -l 50 -w 3 (63). Filtered high-quality reads were assembled using MEGAHIT (version 1.1.2) using the option –min-count 2 –min-contig-len 300 (63). Gene prediction from the assembled contig was predicted using prodigal (version 2.6.3) with -meta mode (63). A total of 5,538,849 genes were identified. A custom script was used to remove incomplete genes, and 1,507,615 complete genes were retained. A nonredundant gene catalog was constructed using CD-HIT (version 4.6.1) with 90% sequence identity and 90% coverage at the protein level (63). High-quality reads were mapped back to the constructed nonredundant gene catalog with 95% identity using SOAPaligner to calculate gene abundance within each sample (66). The clustered amino acid sequences in the gene catalog were then aligned to the GENES database using Diamond (version 0.8.35) to annotate the Kyoto Encyclopedia of Genes and Genomes (KEGG) gene profile (67). The protein sequences were also aligned to the CAZy database using HMMER (version 3.1b2) to annotate the carbohydrate-active enzyme (CAZyme) profile (68). Genes were assigned to the microbial taxa with the highest scores, and the total read count of all genes assigned to a specific taxon was used to estimate its abundance. Normalized RPKM (reads per kilobase of a gene per million mapped reads) values of taxa or KEGG terms were used for statistical analyses.

### Quantitative profiling of BAs.

BAs in colonic digesta were measured as previously described (69). A Waters ACQUITY ultraperformance LC system coupled with a Waters XEVO TQ-S mass spectrometer with an electrospray ionization (ESI) source controlled by MassLynx 4.1 software (Waters, Milford, MA, USA) was used for all analyses. Chromatographic separations were performed with an ACQUITY BEH C_18_ column (1.7 μm, 100 mm by 2.1 mm internal dimensions) (Waters). Ultraperformance liquid chromatography mass spectroscopy (UPLC-MS) raw data obtained with the negative mode were analyzed using TargetLynx applications manager (version 4.1; Waters) to obtain calibration equations and the concentration of each BA in the samples.

### Statistical analysis.

All statistical significance was assessed using a one-way analysis of variance (ANOVA) (SPSS 20 software, IBM, Armonk, NY, USA) except for the intestinal microbiome data. For intestinal microbiome data analysis, α-diversity was analyzed using the Kruskal-Wallis test. PCoA was analyzed using ANOSIM. The relative abundances of the intestinal microbiota and functional genes were analyzed using the Kruskal-Wallis test with false-discovery rate (FDR) adjustment. All results were expressed as the mean ± the standard error of the mean (SEM). Differences were considered statistically significant if *P *was <0.05.

### Data availability.

The bacterial 16S rRNA sequencing data reported in this paper were deposited in the NCBI Sequence Read Archive (SRA) database under accession number PRJNA694703.
