# Supplementary material for: Alginate Alleviates Dextran Sulfate Sodium-Induced Colitis by Promoting Bifidobacterium animalis and Intestinal Hyodeoxycholic Acid Synthesis in Mice
Source: Microbiol Spectr. 2022 Oct 11;10(6):e02979-22. doi: 10.1128/spectrum.02979-22 (PMC9769733; doi:10.1128/spectrum.02979-22)
Supplement: Supplemental file 1 — Supplemental material. Download spectrum.02979-22-s0001.pdf, PDF file, 0.4 MB [file spectrum.02979-22-s0001.pdf]

Supplementary Tables and Figures

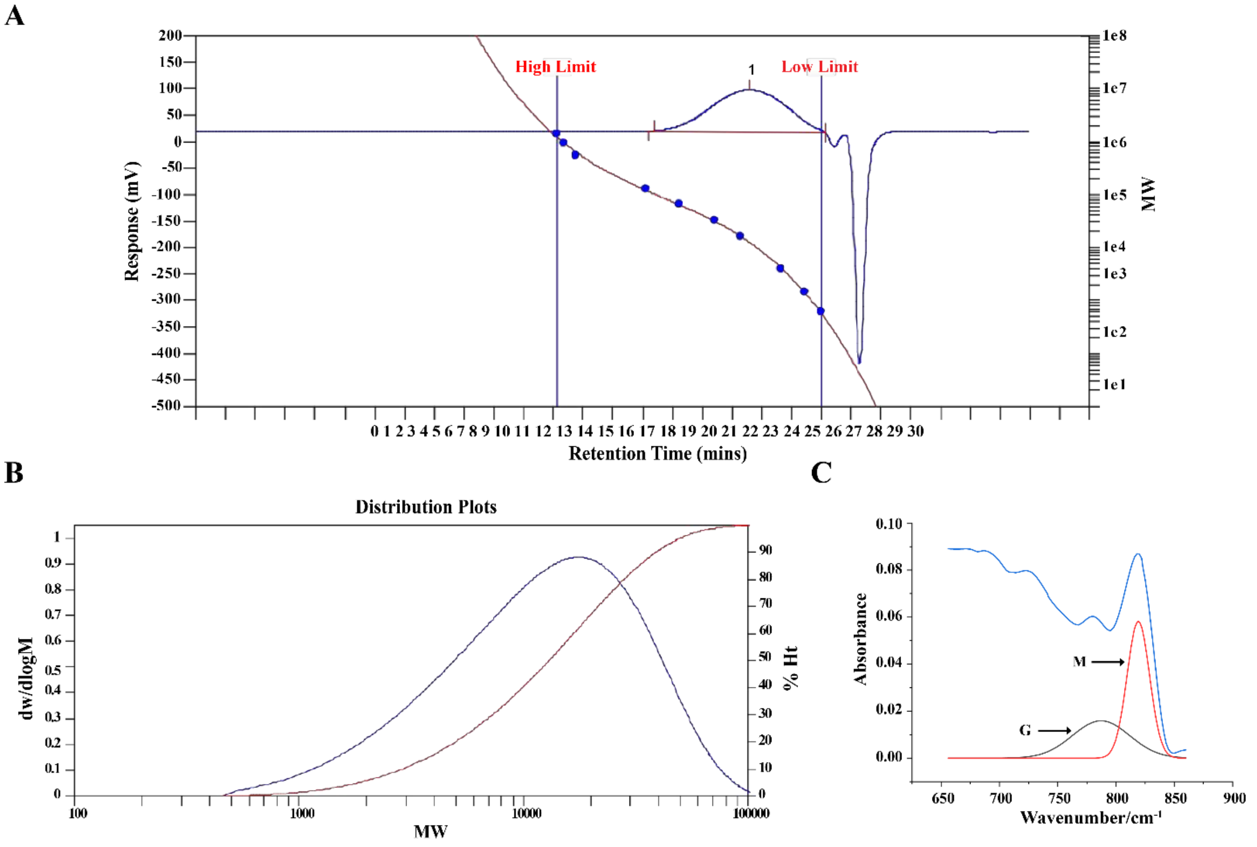

Supplemental Figure S1. Characterization of sodium alginate (ALG). (**A**) GPC Chromatography of ALG. (**B**) The distribution plots of molecular weight of ALG. (**C**) Absorption spectra of ALG by infrared spectra. M and G represent the peak absorption of β-D-mannuronic acid and α-L-guluronic acid, respectively.

Supplemental Table S1. Parameters and criteria of histological damage evaluation[1,2]

Parameters	Score	Histological features
	0	No change
(1) Loss of epithelial surface	1	Localized and mild
(2) Destruction of the crypts	2	Localized and moderate
(3) Infiltration of inflammatory cells	3	Localized and severe
	4	Extensive and moderate
	5	Extensive and severe

[1] Each parameter is scored separately on a scale of 0-5. The histological score is the sum of the scores from all three parameters.

[2] The method was adopted from a previous study with modifications (1).

Supplemental Table S2. Scoring of the disease activity index (DAI)[1,2]

Score	Weight loss (%)	Stool consistency	Fecal blood content
0	None	Normal	Normal
1	0-10		
2	11-15	Loose stool	Occult blood
3	16-20		
4	> 20	Diarrhea	Hemorrhage/Gross bleeding

[1] The DAI score is defined as the sum of three scores from weight loss (%), stool consistency, and fecal blood content.

[2] The method was adopted from a previous study (2).

Supplemental Table S3. Primers used in this study[1]

Target gene	Forward primer (5'-3')	Reverse primer (5'-3')	Annealing temp. (°C)	References
**For host genes of mouse**				
IL-1β	CTCGCAGCAGCACATCAACAAG	GGAAGGTCCACGGGAAAGACAC	60	(3)
TNF-α	TGGGAGTAGACAAGGTACAACCC	CATCTTCTCAAAATTCGAGTGACAA	60	(3)
IL-6	ACCACGGCCTTCCCTACTT	CACAACTCTTTTCTCATTTCCAC	60	(3)
FXR	TGGGCTCCGAATCCTCTTAGA	TGGTCCTCAAATAAGATCCTTGG	60	(4)
TGR5	CCTGGCAAGCCTCATCGTC	AGCAGCCCGGCTAGTAGTAG	60	(4)
β-actin	TGGAATCCTGTGGCATCCATGAAAC	TAAAACGCAGCTCAGTAACAGTCCG	60	(3)
**For bacteria**				
Total bacteria	GTGSTGCAYGGYYGTCGTCA	ACGTCRTCCMCNCCTTCCTC	60	(5)
baiJ	TCAGGACGTGGAGGCGATCCA	TACRTGATACTGGTAGCTCCA	60	(6)
bsh	ATGGGCGGACTAGGATTACC	TGCCACTCTCTGTCTGCATC	54	(7)
7α-HSDH	GGGTATTGTGTATCAAAAGCTGCGG	TCCGTTGCTATAAGCCCAGGTAAGA	60	This study; MH743112.1
7β-HSDH	GTCGTAAAAGCAGACTTTTCGCTGC	TTGATCATTGCCTCATGCTTTTCC	60	This study; KF052988.1

[1] IL-6, interleukin-6; IL-1β, interleukin-1β; TNF-α, tumor necrosis factor-α; FXR, farnesoid X receptor; TGR5, G-coupled protein receptor; *baiJ*, genes encoding bile salt 7α-dehydroxylase; *bsh*, genes encoding bile salt hydrolase; 7α-HSDH, genes encoding 7α-hydroxysteroid dehydrogenase; 7β-HSDH, genes encoding 7β-hydroxysteroid dehydrogenase.

REFERENCES

1. Wang SL, Zhang SY, Huang SM, Wu ZH, Pang JM, Wu YJ, Wang JJ, Han DD. 2020. Resistant maltodextrin alleviates dextran sulfate sodium-induced intestinal inflammatory injury by increasing butyric acid to inhibit proinflammatory cytokine levels. Biomed Res Int 2020: 7694734.
2. Park YH, Kim N, Shim YK, Choi YJ, Nam RH, Choi YJ, Ham MH, Suh JH, Lee SM, Lee CM, Yoon H, Lee HS, Lee DH. 2015. Adequate dextran sodium sulfate-induced colitis model in mice and effective outcome measurement method. J Cancer Prev 20: 260-267.
3. Huang SM, Wu ZH, Liu C, Han DD, Feng CP, Wang SL, Wang JJ. 2019. Milk fat globule membrane supplementation promotes neonatal growth and alleviates inflammation in low-birth-weight mice treated with lipopolysaccharide. Biomed Res Int 2019: 4876078.
4. Sun LL, Xie C, Wang G, Wu Y, Wu Q, Wang XM, Liu J, Deng YY, Xia JL, Chen B, Zhang SY, Yun CY, Lian G, Zhang XJ, Zhang H, Bisson WH, Shi JM, Gao XX, Ge PP, Liu CH, Krausz KW, Nichols RG, Cai JW, Rimal B, Patterson AD, Wang X, Gonzalez FJ, Jiang CT. 2018. Gut microbiota and intestinal FXR mediate the clinical benefits of metformin. Nat Med 24: 1919-1929.
5. Maeda H, Fujimoto C, Haruki Y, Maeda T, Kokeguchi S, Petelin M, Arai H, Tanimoto I, Nishimura F, Takashiba S. 2003. Quantitative real-time PCR using TaqMan and SYBR Green for *Actinobacillus actinomycetemcomitans*, *Porphyromonas gingivalis*, *Prevotella intermedia*, tetQ gene and total bacteria. Fems Immunol Med Mic 39: 81-86.
6. Yoshimoto S, Loo TM, Atarashi K, Kanda H, Sato S, Oyadomari S, Iwakura Y, Oshima K, Morita H, Hattori M, Honda K, Ishikawa Y, Hara E, Honda K. 2013. Obesity-induced gut microbial metabolite promotes liver cancer through senescence secretome. Nature 499: 97-101.
7. Duary RK, Batish VK, Grover S. 2012. Relative gene expression of bile salt hydrolase and surface proteins in two putative indigenous *Lactobacillus plantarum* strains under in vitro gut conditions. Mol Biol Rep 39: 2541-52.
